# Atractylenolide III for central nervous system disorders: a review of multi-target mechanisms and therapeutic potential

**DOI:** 10.3389/fphar.2026.1799976

**Published:** 2026-05-29

**Authors:** Han Li, Ning-Xi Zeng, Lei Ge, Rui-Liang Ye, Yi-Yang Zhuang, Xiao-Na Huang, Qi Liang, Jian V. Zhang, Peng Zhou

**Affiliations:** 1 Shenzhen Bao’an Chinese Medicine Hospital, The Seventh Clinical College of Guangzhou University of Chinese Medicine, Shenzhen, China; 2 Shenzhen Key Laboratory of Metabolic Health, Shenzhen Metabolism and Reproductive Targeted Delivery Proof-of-Concept Center, Shenzhen Institutes of Advanced Technology, Chinese Academy of Sciences, Shenzhen, China; 3 Medical Research Center, Dalang Hospital of Dongguan, Dongguan, China

**Keywords:** *Atractylodes macrocephala* Koidz., central nervous system disorders, natural medicinal metabolites, neuroprotection, review, spleen-fortifying

## Abstract

Central nervous system disorders (CNSDs) are a leading cause of global mortality and disability, yet treatment options remain limited. Natural compounds derived from traditional Chinese herbs constitute a vital resource for discovering novel neuroprotective agents. Atractylenolide III (ATL-III), a sesquiterpene lactone isolated from the spleen-fortifying herb *Atractylodes macrocephala* Koidz., exhibits neuroprotective properties and possesses the ability to cross the blood-brain barrier (BBB). However, a systematic summary of its effects on CNSDs is currently lacking. This review therefore aimed to comprehensively summarize the therapeutic effects and underlying mechanisms of ATL-III against various CNSDs, and to provide insights for future clinical translation. A systematic literature search was conducted using PubMed, Web of Science, Google Scholar, China National Knowledge Infrastructure, and Wanfang Database for studies published or available online between 1 January 2016, and 31 December 2025. The compiled evidence demonstrates that ATL-III is effective in preclinical models of cognitive impairment, cerebral ischemia/reperfusion injury, depressive disorder, and spinal cord injury. Its neuroprotective mechanisms are multifaceted, involving the enhancement of neurotransmitter storage, reduction of neurotoxic protein accumulation, and exerting anti-apoptotic, anti-inflammatory, antioxidant, and autophagy-regulating effects, alongside BBB repair. In conclusion, ATL-III exerts broad neuroprotective effects through multi-target mechanisms, highlighting its promise as a therapeutic candidate for CNSDs. To realize this potential, key future efforts should include: (1) conducting definitive clinical trials to establish its efficacy and safety profile; (2) comprehensively elucidating its pharmacological and toxicological properties; and (3) developing novel delivery strategies to enhance its bioavailability, BBB penetration, and brain retention.

## 1 Introduction

The brain and spinal cord form the central nervous system (CNS), an essential part of the human body. Central nervous system disorders (CNSDs) encompass a group of serious diseases, such as dementia, cognitive dysfunction, cerebrovascular diseases, psychological disorders, and physical injuries to the brain and spinal cord. As primary global contributor to disability and mortality, CNSDs impose a heavy and escalating socioeconomic strain, anticipated to grow with the aging of the global population—a challenge particularly acute in resource-limited developing countries (Feigin and Vos, 2019; Silberberg et al., 2015).

Although research into the pathogenesis and management strategies for CNSDs has yielded progress, effective diagnostic and therapeutic approaches for most CNSDs remain elusive, with current treatments often offering only limited symptomatic relief (Durães et al., 2018; Hussain et al., 2018; Zheng et al., 2026). Consequently, the discovery and development of novel therapeutic agents capable of optimizing existing treatment regimens are of paramount importance.

Natural medicinal metabolites are vital to contemporary medicine and a key source of neuroprotective agents due to their diverse structures, multi-target and multi-pathway actions, and generally safe profiles (Zheng et al., 2026). Traditional Chinese Medicine (TCM) has gathered a wealth of knowledge in the prevention and treatment of CNSDs. Traditional Chinese botanical drugs offer notable benefits due to their holistic approach, targeting multiple aspects with relative safety, effectively addressing both underlying causes and symptoms, and ensuring consistent efficacy with minimal relapse (Alghamdi et al., 2022). Guided by TCM theory, identifying neuroprotective natural medicinal metabolites from relevant botanical drugs has thus become an important pathway in contemporary drug discovery.

Atractylenolide III (ATL-III) is capable of crossing the blood-brain barrier (BBB), its neuroprotective effects have garnered increasing attention. Despite growing interest in the therapeutic potential of ATL-III for CNSDs over the past decade, no review has comprehensively assessed its multifaceted effects. This review therefore undertakes a systematic examination of the literature from the last 10 years, integrating evidence on ATL-III’s role in CNSD prevention and treatment, consolidating its diverse pharmacological mechanisms, and appraising its translational prospects. Ultimately, it aims to offer a structured framework and actionable insights to guide the future development of ATL-III and foster progress in CNSDs therapeutics.

## 2 Source and characteristics of ATL-III

*Atractylodes macrocephala* Koidz (*A. macrocephala*, Baizhu in Chinese), the rhizome of a plant belonging to the Asteraceae family, was first documented in “*The Shennong Materia Medica*” and is also included in the *Chinese Pharmacopoeia* (Chinese Pharmacopoeia Commission, 2025). It is traditionally used to tonify qi, fortify the spleen, dry dampness, promote diuresis, and arrest sweating, primarily for treating digestive disorders such as gastrointestinal ailments, abdominal pain, and diarrhea. *Atractylodes macrocephala* Koidz. contains various metabolites, including lactones, volatile oils, triterpenoids, polysaccharides, phenolic acids, and amino acids.

ATL-III is one of the major active metabolites of *A. macrocephala* Koidz. Its extraction and purification typically involve solvent extraction followed by column chromatography over silica gel and Sephadex, as well as recrystallization. The structural identification is ultimately performed by spectroscopic methods such as mass spectrometry, proton nuclear magnetic resonance, and carbon-13 nuclear magnetic resonance (Chen et al., 2016; Fang et al., 2013; Hai et al., 2023).

ATL-III appears as a white to off-white crystalline powder and belongs to the tricyclic sesquiterpene lactone. Its chemical name is (4aS)-4a,5,6,7,8,8a,9,9a-octahydro-9aβ-hydroxy-3,8aβ-dimethyl-5-methylenenaphtho [2,3-b] furan-2(4H)-one, with a molecular formula of C_15_H_20_O_3_, a relative molecular weight of 248.32, and a CAS number of 73030-71-4. ATL-III is soluble in chloroform, ethyl acetate, and methanol. In terms of chemical stability, ATL-III has a melting point range of 166 °C–169 °C (solvent: chloroform), a boiling point of 424.6 °C ± 45.0 °C at 760 mmHg (National Center for Biotechnology Information, 2026).

## 3 Ethnopharmacological basis of ATL-III in the treatment of CNSDs

“*Huangdi Neijing*” (Yellow Emperor’s Inner Canon) is a foundational classical text of TCM, believed to have been compiled between the Warring States period and the Han Dynasty (approximately 2,000–2,200 years ago). It systematically discusses TCM theories such as yin-yang, five elements, viscera, meridians, etiology, and principles of health preservation and treatment. “*Huangdi Neijing*” highlight that the spleen is crucial for acquired constitution and qi and blood production, while the kidneys are essential for innate constitution, bone health, marrow generation, and brain connection. Additionally, the brain is considered the seat of the original spirit, and the spirit is believed to originate from the essence of water and grains. The TCM theory of “nourishing the innate with the acquired” posits that regulating spleen and stomach function to replenish kidney essence and nourish the brain marrow represents a significant approach for CNSDs prevention and treatment. Therefore, TCM considers the spleen-fortifying therapy as a promising approach for the management of CNSDs.

*Atractylodes macrocephala* Koidz. is one of the most commonly used and well-established spleen-fortifying botanical drugs in China. Clinical studies indicate that Chinese herbal formulas with *A. macrocephala* Koidz., such as *Linggui Zhugan* Decoction (reduces stroke risk in vertigo patients) (Tsai et al., 2016), *Xiaoyao San* Decoction (effective in treating depression) (Wang et al., 2023), and *Jianpi Tianjing Fang* Decoction (may delay conversion from mild cognitive impairment to AD) (He et al., 2008), show significant efficacy in CNSDs treatment.

More than 10 years ago, research demonstrated ATL-III’s neuroprotective effects, showing it significantly improved learning and memory deficits caused by prolonged high-dose homocysteine exposure and inhibited homocysteine-induced apoptosis in primary neurons (Zhao et al., 2015). ATL-III safeguarded primary mouse cortical neurons from glutamate-induced damage (Liu et al., 2014) and offered different levels of protection to PC12 cells against hypoxia, excitatory amino acid, and high-calcium injuries (Luo and Sun, 2012). The structural resemblance between the B-C ring of ATL-III and serotonin indicates its potential role as a partial antagonist at serotonin receptors (Murayama et al., 2014).

## 4 Review methodology

A comprehensive literature review was conducted using Web of Science, PubMed, Google Scholar, China National Knowledge Infrastructure (CNKI), and Wanfang Database. The search strategy employed the term (TS = “Atractylenolide III” OR “Atractylenolide iii”) to identify relevant studies. Subsequently, three authors independently screened the retrieved records manually based on titles, abstracts, and full texts to determine whether the studies addressed the effects of ATL-III on CNSDs.

This review included studies based on these criteria: (1) clinical or preclinical studies assessing ATL-III’s therapeutic efficacy against CNSDs; (2) studies published or available online between 1 January 2016, and 31 December 2025 (the past decade).

Exclusion criteria included: (1) studies where CNSDs were secondary or comorbid; (2) absence of biochemical analyses; (3) publications not in Chinese or English; (4) types of publications like types like meta-analyses, clinical reports, reviews, dissertations, or conference abstracts; (5) *in vitro* experiments not using CNS-related cells or cell lines (e.g., neurons, glial cells, neural stem cells, neuron-like cells).

## 5 Results

Within the time frame from 1 January 2016, to 31 December 2025, the search results from each database were as follows: Web of Science, 181 records; PubMed, 192 records; Google Scholar, 166 records; CNKI, 247 records; and Wanfang Data, 189 records.

After removing duplicate records across databases and screening according to the inclusion and exclusion criteria, a total of 21 preclinical studies were included in this review. Among these, 14 involved *in vivo* studies (Table 1) and 14 involved *in vitro* studies (Table 2); no clinical studies were included.

**TABLE 1:** Summary of *in vivo* experiments on atractylenolide III against central nervous system disorders.

Disease	Dosage	Inducement	Animal	Therapeutic effect after treatment	Molecular mechanism after treatment	Pharmacological effects	References
Cognitive impairment	30 mg/kg/day. Intraperitoneal injection for 36 days	Intracerebroventricular injection of Aβ_1-42_	Male C57BL/6 mouse	Barnes maze test the time spent in seeking the escape box ↓; accuracy in locating the escape hole during the probe test Morris water maze test: the time spent in seeking the platform ↓; the time in the target quadrant ↑; the times of crossing platform ↑. Electrophysiological recordings excitatory postsynaptic potentials ↑	ROS ↓, H_2_O_2_ ↓, i-NOS ↓ in hippocampus.Nrf2 ↑, HO-1 ↑, SOD-1 ↑ in hippocampus	Inhibition of oxidative stress	Yuan et al. (2025)
Cognitive impairment	15 and 30 mg/kg/day. Intragastric administration for 8 weeks	Transgene	Male APP/PS1 mouse	Morris water maze test: the time spent in seeking the platform ↓; the time in the target quadrant ↑; the percentage of time in the target quadrant ↑ the times of crossing platform ↑. Y maze test: the ratio of spontaneous alternation Novel object recognition test: discrimination index ↑	Aβ plaques ↓ in hippocampus. P62 ↓, LC3 II↑, LAMP2 ↑ in cortex and hippocampus	Inhibition of Aβ deposition Regulation of autophagy	Zhang et al. (2025b)
Cognitive impairment	2.4 mg/kg/day. Intragastric administration for 8 weeks	Intracerebroventricular injection of Aβ_1-42_	Female and Male C57BL/6 mouse	Morris water maze test: the time spent in seeking the platform ↓ the times of crossing platform ↑	Aβ_1-42_ ↓ and AQP4 ↑ in brain	Inhibition of Aβ deposition	Hao et al. (2025)
Cognitive impairment	0.6, 1.2 and2.4 mg/kg/day. Intragastric administration for 6 weeks	Intracerebroventricular injection of streptozotocin	Male Sprague-Dawley rats	Open field test the total travelled distance ↑ the mean speed ↑ the number of entries in the central area ↑ the time spent in central area ↑. Novel object recognition test: discrimination index Morris water maze test:	Bcl-2 ↑ and Bax ↓ in hippocampus p-Tau ↓ in hippocampus p-PI3K ↑, p-Akt ↑ and GSK-3β ↓ in hippocampus	Inhibition of Tau phosphorylation Anti-apoptosis	Liu et al. (2024)
​	​	​	​	the time spent in seeking the platform ↓ the time in the target quadrant ↑ the times of crossing platform ↑	​	​	​
Cognitive impairment	0.6 and 1.2 mg/kg/day. Intragastric administration for 4 weeks	Intracerebroventricular injection of Aβ_1-42_	Male Sprague-Dawley rats	Morris water maze test: the time spent in seeking the platform ↓ the time in the target quadrant ↑	Bcl-2 ↑ in hippocampus.AchE ↓ in hippocampus	Amelioration of cholinergic dysfunction Anti-apoptosis	Yu et al. (2018)
Cognitive impairment	1.2, 2.4 and 4.8 mg/kg. Intragastric administration for once	Isoflurane-induced	Male Sprague-Dawley rats	​	The number of apoptosis cells ↓ in hippocampus Pathological scores ↓ in hippocampal HE staining.Caspase-3 ↓, Bcl-2 ↑ and Bax ↓ in hippocampus.LC3 II/LC3 I ↓, Beclin-1 ↓ and p62 ↑ in hippocampus.TNF-α ↓, IL-1β ↓ and IL-6 ↓ in serum and hippocampus p-PI3K ↑, p-Akt ↑, p-mTOR ↑ and p-NF-κB ↓ in hippocampus	Regulation of autophagy Anti-apoptosis Anti-inflammation	Zhu et al. (2021)
Cerebral ischemia/reperfusion injury	10, 20 and 40 mg/kg/day. Pretreat with Intraperitoneal injection for 7 days	Middle cerebral artery occlusion/reperfusion	Male Kunming mouse	Neurological function test: modified neurological severity scores ↓ 0.2,3,5-triphenyltetrazolium chloride staining: infarct volume ↓. Grip strength test: hanging times ↑	Pathological scores ↓ in hippocampal HE and Nissl staining The number of apoptosis cells ↓ in hippocampal CA1 area Bcl-2 ↑ and Bax ↓ in hippocampus 4-HNE ↓, Keap1 ↓, HO-1↑ and Nrf2 ↑ in hippocampus	Inhibition of oxidative stress Anti-apoptosis	Guo et al. (2025)
Cerebral ischemia/reperfusion injury	10 and 20 mg/kg/day. Intragastric administration for 7 days	Middle cerebral artery occlusion/reperfusion	Male Sprague-Dawley rats	Neurological function test: neurological severity scores ↓ 2,3,5-triphenyltetrazolium chloride staining: infarct volume ↓.Evans	Pathological scores ↓ in hippocampal HE staining Neuronal density ↑ in hippocampal Nissl staining ZO-1 ↑, Occludin ↑ and	Repairment of blood-brain barrier Anti-inflammation	Mao et al. (2026)
​	​	​	​	blue test: blood–brain barrier leakage ↓.Brain water content ↓	Claudin 1 ↑ in hippocampus p-PI3K ↑ and p-Akt ↑ in hippocampus p-NF-κB p65↓, NLRP3 ↓, IL-1β ↓, IL-6 ↓ and TNF-α ↓ in hippocampus	​	​
Cerebral ischemia/reperfusion injury	0.1, 1 and 10 mg/kg/day. Intravenously injection for beginning of ischemia (0 h) and the onset of reperfusion (1 h), and every day after reperfusion for 7 days	Middle cerebral artery occlusion/reperfusion	Male C57BL/6 J mouse	Neurological function test: neurological severity scores ↓ 2,3,5-triphenyltetrazolium chloride staining: infarct volume ↓.Cerebral blood flow ↑.Brain water content ↓	IL-1β ↓, IL-6 ↓, TNF-α ↓, IL-10 ↑, Arg-1 ↑, and CD206 ↑ in ischemic penumbra cortex. p-JAK2 ↓ and p-STAT3↓ in microglia of ischemic penumbra cortex Drp1 ↓ in microglia, astrocytes, and neurons of ischemic penumbra cortex	Anti-inflammation Inhibition of mitochondrial fission	Zhou et al. (2019)
Cerebral ischemia/reperfusion injury	45 and 90 mg/kg/day. Pretreat with Intraperitoneal injection for 3 days	Middle cerebral artery occlusion/reperfusion	Male Sprague-Dawley rats	Neurological function test: neurological severity scores ↓ 2,3,5-triphenyltetrazolium chloride staining: infarct volume ↓	The number of apoptosis cells ↓ in hippocampus. TNF-α ↓, IL-6 ↓ and IL-10 ↑ in serum CHOP ↓, Caspase-3 ↓, Caspase-12 ↓ and Bcl-2 ↑ in hippocampus p-PERK/PERK ↓, p-eIF2α/eIF2α ↓ and ATF4 ↓ in hippocampus	Anti-apoptosis Inhibition of endoplasmic reticulum stress	Gao et al. (2024)
Cerebral ischemia/reperfusion injury	0.5, 1 and 2 mg/kg/day. Intragastric administration for 4 weeks	Middle cerebral artery occlusion/reperfusion	Male Sprague-Dawley rats	Neurological function test: neurological severity scores ↓ Balance beam test score ↓0.2,3,5-triphenyltetrazolium chloride staining: infarct volume ↓.Brain water content ↓	Pathological scores ↓ in ischemic hemisphere HE staining The number of Nissl substance ↑ in ischemic hemisphere Shh ↑ and Ptch1 ↑in ischemic hemisphere	Anti-apoptosis Anti-inflammation	Zhou and Lu (2024)
Cerebral ischemia/reperfusion injury	30 and 90 mg/kg/day. Intragastric administration for 15 days	Middle cerebral artery occlusion/reperfusion	Male Sprague-Dawley rats	Neurological function test: neurological severity scores ↓ 2,3,5-triphenyltetrazolium chloride staining: infarct volume ↓	The number of apoptosis cells ↓ in brain TNF-α ↓, IL-6 ↓ and IL-1β ↓ in serum SOD ↑, CAT ↑ and MDA ↓ in brain Bax ↓, Bcl-2 ↑, SIRT1 ↑ and Nrf2↑ in brain	Inhibition of oxidative stress Anti-apoptosis	Zhang et al. (2024)
Spinal cord injury	5 mg/kg/day. Intragastric administration for 42 days	Strike the T9 spinal cord	Female Sprague-Dawley rats	Basso-Beattie-Bresnahan locomotion rating scale score ↑ Grid walking test: the rate of footfalls on the grid ↓ Footprint analysis score↑	Lesion areas ↓ in spinal HE staining Residual myelination↑ in spinal LFB staining Number of motor neurons ↑ in spinal Nissl staining The numbers of activated microglia/macrophages ↓ in spinal The proportion of M1 cells ↓ and M2 cells↑ in spinal p-IκBα ↓, p-p65 ↓, p-p38 ↓, p-JNK ↓ and p-Akt ↑ in spinal iNOS ↓, Arg-1 ↑, TNF-α ↓, IL-1β ↓, IL-6 ↓ and IL-10 ↑ in spinal	Anti-inflammation	Xue et al. (2022)
Depressive disorder	30 mg/kg/day. Intragastric administration for 28 days	LPS-induced and CUMS	Male Sprague-Dawley rats	LPS-induced and CUMS Sucrose preference test: sucrose preference ↑ Novelty-suppressed feeding test: latency to feeding ↓ Forced swimming test: immobility ↓	CUMS:TNF-α ↓, IL-1β ↓ and IL-6 ↓ in hippocampus	Anti-inflammation	Zhou et al. (2021)

**TABLE 2:** Summary of *in vitro* experiments on atractylenolide III against central nervous system disorders.

Dosage	Inducement	Cell type	Molecular mechanism after treatment	Pharmacological effects	References
5, 10, and 20 µM for 24 h	APP_SWE_	SH-SY5Y	p62 ↓, LC3-II ↑, LAMP2 ↑, TFEB ↑ and YY1 ↑	Enhancement of autophagy	Xue et al. (2022)
1, 10, 50, 100 and 200 μM for 48 h	Aβ_1-42_	C8-D1A	Aβ_1-42_ ↓ and AQP4 ↑	Inhibition of Aβ deposition	Yuan et al. (2025)
1 μM for 24 h after pretreat 2 h	LPS	BV-2	TNF-α ↓, iNOS ↓, MyD88 ↓, TLR4 ↓, COX-2 ↓, NO ↓ and NF-κB p65 ↓	Inhibition of oxidative stress Anti-inflammation	Zhang et al. (2025b)
20 mM for 6 h reoxygenation phase	OGD/R	HT22	Bcl-2 ↑ and Bax ↓.ROS↓, MDA↓, SOD ↑, GSH↑, Keap ↓, HO-1↑ and Nrf2 ↑	Inhibition of oxidative stress Anti-apoptosis	Hao et al. (2025)
50, 100, 150, 200, and 250 μM for 24 h reoxygenation phase	OGD/R	PC12	Apoptotic cells rate ↓.LDH ↓, SOD ↑, MDA ↓ and ROS ↓.NLRP3 ↓, IL-1β ↓, IL-6 ↓ and TNF-α ↓. p-PI3K ↑, p-Akt ↑ and p-NF-κB p65↓	Inhibition of oxidative stress Anti-apoptosis.Anti-inflammation	Yi et al. (2021)
0.01, 0.1, and 1 µM for 48 h	OGD/R	Primary microglia	IL-1β ↓, IL-6 ↓, TNF-α ↓, IL-10 ↑, Arg-1 ↑, and CD206 ↑. p-JAK2 ↓ and p-STAT3↓ p-Drp1 ↓ in cytoplasm and mitochondria Fragmented mitochondria↓	Anti-inflammation Inhibition of mitochondrial fission	Guo et al. (2025)
15, 30, and 60 μM for 24 h reoxygenation phase	OGD/R	HT22	Apoptotic cells rate ↓.IL-1β ↓, TNF-α ↓, IL-10 ↑ and TGF-β ↑.Fe^2+^ ↓, SOD ↑, MDA ↓ and ROS ↓.Shh ↓, Gli1 ↓, Bcl-2 ↑ and Bax ↓	Inhibition of oxidative stress Anti-apoptosis Anti-inflammation	Mao et al. (2026)
5, 10, 20, and 40 μM for 6 h reoxygenation phase	OGD/R	HT22	Apoptotic cells rate ↓.IL-1β ↓, IL-6 ↓ and TNF-α ↓.GSH ↑, SOD ↑, MDA ↓ and ROS ↓.Caspase-3 ↓, Bcl-2 ↑ and Bax ↓	Inhibition of oxidative stress Anti-apoptosis Anti-inflammation	Zhou et al. (2019)
1, 10 and 20 μM for 2-h pretreatment following 24-h treatment	Corticosterone-injured	PC12	Apoptotic cells rate ↓.TNF-α ↓, LDH ↓ and Ca^2+^ ↓.Mitochondrial membrane potential ↑.Caspase-3 ↓, CytC ↓, Bcl-2 ↑ and Bax ↓. p-ERK1/2 ↓, p-p38 ↓ and p-JNK ↓.NF-κB p65↓ and IκBα ↑	Anti-apoptosis Anti-inflammation	Han et al. (2025)
25, 50, and 100 µM for 24 h	LPS-induced	BV2	IL-1β ↓, IL-6 ↓, p-IκBα ↓ and p-p65 ↓PGC-1α ↑ and COX4 ↑.Mitochondrial membrane potential ↑	Improvement of mitochondrial function Anti-inflammation	Guo et al. (2024)
①1, 10 and 100 μM for 1, 3 and 6 h without modeling. ②100 μM 3-h pretreatment following 100 μM 3-h treatment ③100 μM 3-h pretreatment following 100 μM 15-, 30-, and 60- min treatment	LPS-induced	RAW264.7, MG6 and primary microglia	①TLR4↓ in MG6 cells②IL-1β ↓, IL-6 ↓, TNF-α ↓, i-NOS ↓ and COX-2 ↓ in RAW264.7 cells, MG6 cells, and primary microglia ③p-p38 MAPK ↓, p-JNK ↓, and p-NF-κB ↓ in MG6 cells	Anti-inflammation	Cai et al. (2025)
35 μM for 2-h pretreatment following 12-h treatment	LPS-induced	BV2 cell	IL-1β ↓, IL-6 ↓ and TNF-α ↓.LC3II ↑ and p62 ↓	Anti-inflammation Enhancement of autophagy	Novianti et al. (2021)
1, 10, and 100 µM for 24 h	LPS-induced	BV-2	iNOS ↓, Arg-1 ↑, TNF-α ↓, IL-1β ↓, IL-6 ↓ and IL-10 ↑ p-IκBα ↓, p-p65 ↓, p-p38 ↓, p-JNK ↓ and p-Akt ↑	Anti-inflammation	Gong et al. (2019)
20, 40, 80, and 160 µM for 24 h	H_2_O_2_-induced	Neuro-2A	ROS ↓, H_2_O_2_ ↓, i-NOS ↓, T-AOC ↑ and T-SOD ↑	Inhibition of oxidative stress	Zhou et al. (2023)

To enable a rigorous assessment of the evidence, we performed a scoring of the 21 included studies (Table 3) based on predefined criteria adapted and modified from SYRCLE’s risk of bias tool. The following factors were assessed: mutual validation between *in vivo* and *in vitro* experiments, peer review status, random allocation, blinded outcome assessment, adequacy of sample size, declaration of conflicts of interest, relevance of the model to the disease condition, use of multiple dose levels, inclusion of positive/negative controls, co-administration of agonists/inhibitors.

**TABLE 3:** Evidence scores of including 21 studies.

References	*In vivo* combining *in vitro*	Peer reviewed publication	Random allocation to group	Blinded assessment of outcome	Sample size calculation	Statement of a potential conflict of interest	Appropriate model	Multi-doses	Positive or negative controls	Drug combining agonists or inhibitors	Total score
Cai et al. (2025)	​	√	√	​	​	√	√	√	√	​	6
Gao et al. (2024)	​	√	√	​	​	√	√	√	​	√	6
Gong et al. (2019)	​	√	√	​	​	√	√	√	​	​	5
Guo et al. (2024)	​	√	√	​	​	√	√	√	√	​	6
Guo et al. (2025)	√	√	√	​	​	√	√	√	√	√	8
Han et al. (2025)	​	√	√	​	​	√	√	√	√	​	6
Hao et al. (2025)	√	√	√	​	​	√	√	​	​	√	6
Liu et al. (2024)	​	√	√	​	​	√	√	√	√	​	6
Mao et al. (2026)	√	√	√	​	​	√	√	√	√	√	8
Novianti et al. (2021)	​	√	√	​	​	√	√	​	​	​	4
Xue et al. (2022)	√	√	√	​	​	√	√	√	​	​	6
Yi et al. (2021)	​	√	√	​	​	√	√	√	√	​	6
Yu et al. (2018)	​	√	√	​	​	√	√	√	​	​	5
Yuan et al. (2025)	√	√	√	​	​	√	√	√	​	​	6
Zhang et al. (2024)	​	√	√	​	​	√	√	√	​	√	6
Zhang et al. (2025b)	√	√	√	​	​	√	√	√	√	√	8
Zhou and Lu (2024)	​	√	√	​	​	√	√	√	​	√	6
Zhou et al. (2019)	√	√	√	​	-	√	√	√	√	√	8
Zhou et al. (2021)	​	√	√	√	​	√	√	√	​	​	6
Zhou et al. (2023)	​	√	√	​	​	√	√	​	​	​	4
Zhu et al. (2021)	​	√	√	​	​	√	√	√	​	​	5

**FIGURE 1:**
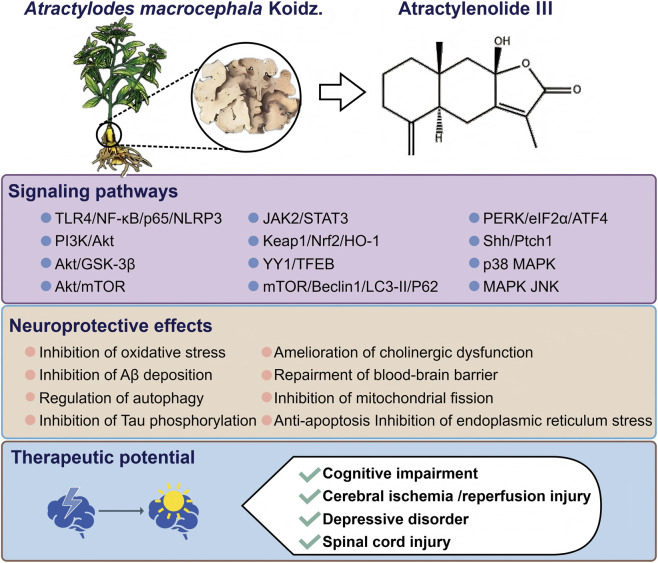
Atractylenolide III mediates multiple neuroprotective effects via multiple signaling pathways and exhibits therapeutic potential in various central nervous system.

The above studies indicate that ATL-III exhibits therapeutic potential against various central nervous system diseases through multi-target and multi-mechanism actions (Figure 1).

### 5.1 Effects of ATL-III on cognitive impairment models

**FIGURE 2:**
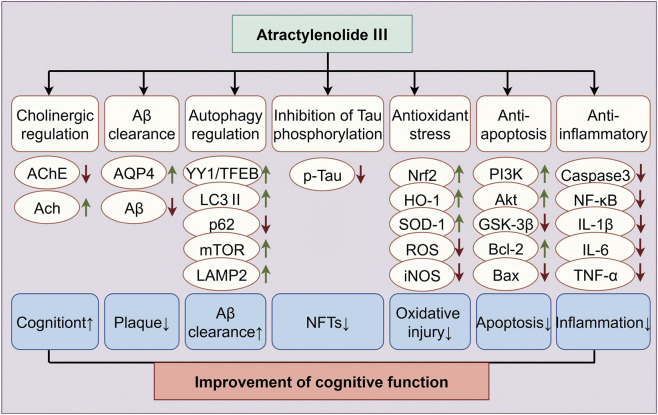
Effects of ATL-III on cognitive impairment models.

Alzheimer’s disease (AD) is a neurodegenerative disorder of the central nervous system, characterized by worsening cognitive impairment. The impact of ATL-III on cognitive impairment is shown in Figure 2. With increasing societal aging, AD incidence is rising annually, notably in developing countries including China. AD represents a major challenge to healthcare resources and creates a significant socioeconomic burden. The pathogenesis of AD is not well-defined; however, the development of AD is thought to involve several key theories, encompassing the amyloid-beta (Aβ) cascade, hyperphosphorylation of Tau protein, dysfunction of the cholinergic system, neuronal and synaptic damage, and inflammation. Currently, there are no disease-modifying drugs for AD. First-line clinical agents like donepezil and memantine neither affect life expectancy nor alter the overall disease course. Furthermore, their use carries risks of hepatotoxicity and gastrointestinal adverse effects (Li et al., 2024). Faced with the ineffectiveness and severe side effects of many new drug candidates over the last 20 years, the field has turned to natural compounds as potential sources for developing AD treatments. This has led to a renewed focus on exploring these alternatives for viable therapeutic strategies.

The dysfunction of central cholinergic systems is a foundational and widely endorsed theory (Baviskar and Mahajan, 2026). Acetylcholine (ACh) interacts with cholinergic receptors, crucial for neuron activation, cognitive function, and memory enhancement. After impulse transmission, ACh is created by choline acetyltransferase and subsequently degraded by acetylcholinesterase (AChE) to cease its activity. In contrast to healthy individuals who have enough ACh production, AD patients face a significant loss of cholinergic neurons (Li et al., 2022). AChE inhibitors continue to be fundamental in the clinical management of AD (Li et al., 2022). Yu et al. (2018) (Yu et al., 2018) demonstrated that oral ATL-III reduced hippocampal AChE expression and enhanced cognitive function in an AD rat model induced by Aβ_1-42_ intracerebroventricular injection.

AD is characterized by Aβ plaque deposition and neurofibrillary tangles from hyperphosphorylated Tau protein. Aβ has a pronounced tendency to aggregate. The toxic small aggregates and insoluble deposits that form initiate a series of pathological events, resulting in synaptic impairment and neuronal death, which ultimately causes permanent brain damage (Hein et al., 2025). While not solely responsible for all neuronal damage in AD, the deposition of Aβ is a pivotal trigger that activates multiple disease-related pathways (Li et al., 2022). Given its role, strategies aimed at attenuating Aβ generation and augmenting its removal remain cardinal in the pursuit of anti-AD therapeutics (Keil et al., 2025). Aquaporin 4 (AQP4), a selective water channel in astrocyte membranes, is crucial for astrocyte functions, including Aβ phagocytosis. AQP4 deficiency impairs astrocytic uptake of Aβ_1-42_, reduces Aβ clearance, and facilitates plaque formation (Feng et al., 2023). Evidence indicates that oral ATL-III enhances AQP4 expression and reduces Aβ levels in the brains of AD mice injected with Aβ_1-42_, leading to improved cognitive function (Hao et al., 2025). Concurrent *in vitro* experiments showed ATL-III alleviated Aβ_1-42_-induced damage to C8-D1A astrocytes, partly by upregulating AQP4 (Hao et al., 2025). Autophagy is a crucial lysosome-mediated degradation process that preserves cellular homeostasis by removing damaged organelles and abnormal protein aggregates (Yang et al., 2025). In AD pathogenesis, reduced autophagy leading to amyloid plaque formation significantly exacerbates pathology and worsens disease features (Hussain et al., 2025). Study shows that oral ATL-III administration boosts autophagy, facilitates Aβ clearance, and enhances cognition in amyloid precursor protein (APP)/presenilin 1 (PS1) transgenic AD model mice (Zhang X. et al., 2025). This is achieved by upregulating lysosome-associated membrane protein 2 (LAMP2) and microtubule-associated protein one light chain 3-II (LC3-II) expression in the hippocampus and cortex, and downregulating P62 (Zhang X. et al., 2025). *In vitro* experiment further confirmed ATL-III promoted autophagy and Aβ clearance in SH-SY5Y APP_SWE_ cells by upregulating Yin Yang 1 (YY1) protein and transcription factor EB (TFEB), leading to increased LAMP2 and LC3-II and decreased P62 expression (Zhang X. et al., 2025). Neurofibrillary tangles, formed by hyperphosphorylated Tau, results in the loss of synapses and dysfunction of neurons (Lu et al., 2026). The positive correlation between Tau phosphorylation levels and AD severity highlights the importance of targeting excessive Tau phosphorylation as a treatment approach (Therriault et al., 2025). Studies show oral ATL-III reduced hippocampal Tau phosphorylation levels in an AD rat model induced by intracerebroventricular streptozotocin (STZ) injection (Liu et al., 2024).

The brain, due to its high metabolic activity, generates substantial oxidative products but possesses relatively low intrinsic antioxidant capacity, making it susceptible to oxidative stress (Yu et al., 2024). In AD, oxidative stress contributes to Aβ production, is associated with Tau hyperphosphorylation, and directly causes significant neuronal damage (Dhapola et al., 2024). Therefore, suppressing oxidative stress by elevating antioxidants or reducing pro-oxidants may constitute a therapeutic strategy for AD. Research by Yuan et al. (2025) (Yuan et al., 2025) indicates that intraperitoneal injection of ATL-III mitigated oxidative stress and enhanced cognitive function in the hippocampus of AD mice induced by intracerebroventricular injection of Aβ_1-42_. This effect was achieved by upregulating nuclear factor erythroid 2-related factor (Nrf2), heme oxygenase-1 (HO-1), and superoxide dismutase 1 (SOD-1), while decreasing reactive oxygen species (ROS), H_2_O_2_, and inducible nitric oxide synthase (iNOS) production (Yuan et al., 2025).

Current anti-AD therapies are limited by their failure to address the widespread loss of neurons and synapses in AD brains (Se Thoe et al., 2021). Research suggests that oral ATL-III reduces hippocampal cell apoptosis and enhances cognitive function in AD rat model induced by intracerebroventricular STZ injection (Liu et al., 2024). This effect led to enhanced phosphorylation of phosphatidylinositol 3-kinase (PI3K) and protein kinase B (Akt), reduced phosphorylation of glycogen synthase kinase-3β (GSK-3β), resulting in increased B-cell lymphoma-2 (Bcl-2) expression and decreased Bcl-2 associated X protein (Bax) expression (Liu et al., 2024). Yu et al. (2018) (Yu et al., 2018) found that oral ATL-III increased hippocampal Bcl-2 expression and inhibited apoptosis in an AD rat model induced by Aβ_1-42_ intracerebroventricular injection.

Furthermore, while anesthetic use alleviates intraoperative discomfort, excessive administration may cause neurological damage, including neuronal injury and neuroinflammation. However, almost all commonly used intravenous and inhalational anesthetics can induce neuronal death and neurotoxicity *in vitro* (Shao et al., 2019). Research shows that oral ATL-III reduces isoflurane-induced hippocampal neuronal apoptosis by modulating cysteinyl aspartate-specific proteinase 3 (Caspase-3), Bax, and Bcl-2 levels; decreases excessive autophagy by adjusting the LC3-II/LC3-I ratio, Beclin-1, and P62; and diminishes neuroinflammation by inhibiting nuclear factor kappa-B (NF-κB) and lowering pro-inflammatory cytokines such as tumor necrosis factor α (TNF-α), interleukin-1β (IL-1β), and interleukin-6 (IL-6) (Zhu et al., 2021). The activation of the PI3K/Akt/mammalian target of rapamycin (mTOR) pathway was linked to protective effects against isoflurane-induced apoptosis, autophagy, and inflammation (Zhu et al., 2021).

### 5.2 Effects of ATL-III on cerebral ischemia/reperfusion injury models

**FIGURE 3:**
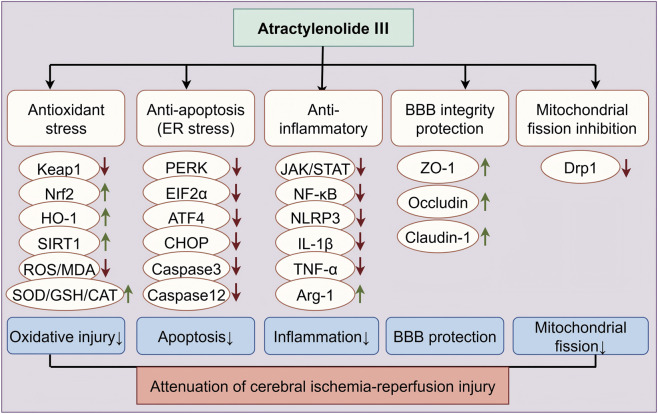
Effects of ATL-III on cerebral ischemia/reperfusion injury models.

In China, stroke represents a critical public health challenge for the adult population. It is marked by high incidence, significant disability and mortality rates, frequent recurrence, and considerable economic costs. These factors collectively establish stroke as a paramount concern and a leading cause of premature mortality and disability nationwide (Stroke Prevention Project and Ji, 2025). Stroke can be categorized into ischemic and hemorrhagic types. Ischemic stroke, encompassing cerebral embolism, thrombosis, and lacunar infarction, constitutes more than 80% of cases (Li et al., 2021). First-line pharmacological reperfusion strategies for ischemic stroke include thrombolysis, anticoagulation, antiplatelet, and fibrinolytic therapies. A core objective of these approaches is to improve cerebral hemodynamics and decrease edema formation. While reperfusion is essential, it can paradoxically exacerbate neuronal damage, leading to cerebral ischemia/reperfusion injury (CIRI) (Li et al., 2021). The complexity of the pathological process in ischemic stroke renders single-target drugs insufficient to fully interrupt the CIRI cascade, pointing to the potential effectiveness of drugs with multiple function. The effect of ATL-III on CIRI is shown in Figure 3.

CIRI triggers significant ROS production, causing lipid peroxidation, protein oxidation, and DNA damage (Guo et al., 2024). 4-hydroxynonenal (4-HNE), a byproduct of lipid peroxidation, exacerbates oxidative stress. The Nrf2/HO-1 signaling pathway is crucial for maintaining cellular redox balance within endogenous antioxidant defense mechanisms. In typical circumstances, Kelch-like ECH-associated protein 1 (Keap1) holds Nrf2 in the cytoplasm, promoting its ubiquitination and later degradation by the proteasome; in the presence of oxidative or electrophilic stress, Nrf2 detaches from Keap1, moves into the nucleus, and binds to the antioxidant response element, initiating the transcription of genes like SOD and HO-1 that offer cellular protection (Sun et al., 2023). In a mouse model of middle cerebral artery occlusion/reperfusion (MCAO/R), intraperitoneal administration of ATL-III alleviated hippocampal oxidative stress and decreased cerebral infarct size by reducing 4-HNE and Keap1 levels, while enhancing Nrf2 and HO-1 expression; concurrent *in vitro* experiments demonstrated that ATL-III mitigated oxidative stress in oxygen-glucose deprivation/reoxygenation (OGD/R)-treated HT22 cells by downregulating Keap1, activating the Nrf2/HO-1 pathway, reducing ROS and malondialdehyde (MDA) levels, and enhancing SOD and glutathione (GSH) (Guo et al., 2025). Sirtuin 1 (SIRT1) is a deacetylase that regulates the activity of its target protein Nrf2 (Zhang et al., 2021). In an MCAO/R rat model, oral ATL-III inhibited cerebral oxidative stress and reduced infarct area by activating the SIRT1/Nrf2 pathway, decreasing MDA, and increasing SOD and catalase (CAT) (Zhang et al., 2024). Additionally, multiple *in vitro* studies indicate ATL-III alleviated oxidative stress damage in OGD/R-treated HT22 and PC12 cells, reducing ferrous ion, lactate dehydrogenase (LDH), ROS, and MDA levels while increasing SOD and GSH (Guo et al., 2024; Han et al., 2025; Mao et al., 2026).

Oxidative stress can exacerbate brain injury by inducing apoptosis during ischemic stroke (Pawluk et al., 2024). A wealth of data *in vivo* and *in vitro* researches suggest that ATL-III inhibits apoptosis in the brains of MCAO/R animal models and OGD/R cell models (Guo et al., 2024; Guo et al., 2025; Han et al., 2025; Mao et al., 2026; Zhang et al., 2024). Notably, research indicates ATL-III can inhibit endoplasmic reticulum (ER) stress in the hippocampus of MCAO/R rats. ER stress is a cellular self-protective mechanism to alleviate unfolded protein concentration, preventing aggregation. However, during ischemic stroke, excessive ER stress can trigger the ER apoptosis pathway, promoting cell death (Wang L. et al., 2022). The PKR-like endoplasmic reticulum kinase (PERK)/eukaryotic initiation factor 2α (eIF2α)/activating transcription factor 4 (ATF4) signaling pathway is an ER stress-induced apoptotic mechanism. PERK, a transmembrane sensor on the ER, phosphorylates eIF2α during ER stress, promoting ATF4 protein expression. ATF4 modulates C/EBP-homologous protein (CHOP) expression, enhancing apoptosis through Caspase-3 and cysteinyl aspartate-specific proteinase 12 (Caspase-12) activation (Luo et al., 2023). Research by Gao et al. (2024) (Gao et al., 2024) indicates that ATL-III suppresses the PERK/eIF2α/ATF4 signaling pathway, leading to decreased expression of CHOP, Caspase-3, and Caspase-12, reduced apoptosis, and a smaller infarct area in the hippocampus of MCAO/R rats.

Mitochondrial dysfunction is particularly important in ischemic stroke. Excessive activation of dynamin-related protein 1 (Drp1), which facilitates mitochondrial fission, is crucial in causing mitochondrial dysfunction and intensifying apoptosis (Jia et al., 2025). Research indicates tail vein injection of ATL-III downregulated Drp1 expression in the hippocampus of MCAO/R mice, inhibited mitochondrial fission, and reduced infarct area; concurrent *in vitro* experiments showed ATL-III downregulated Drp1 in OGD/R-treated primary microglia, inhibiting mitochondrial fission (Zhou et al., 2019).

Neuroinflammation is a significant mechanism of post-stroke brain injury, exacerbating damage not only through direct neuronal injury but also by disrupting BBB integrity (Lu and Wen, 2024). The Janus kinase (JAK)/signal transducer and activator of transcription (STAT) pathway is crucial in microglia for cytokine production, immune cell recruitment and activation, and adaptive immune response initiation. However, its overactivation may lead to the release of pro-inflammatory factors and neuronal apoptosis (Liang et al., 2016; Song et al., 2023). Research indicates that administering ATL-III via tail vein injection in an MCAO/R mouse model inhibits the JAK/STAT pathway in the ischemic penumbra cortex, leading to decreased levels of pro-inflammatory cytokines IL-1β, IL-6, and TNF-α, and increased levels of anti-inflammatory cytokines CD206, IL-10, and arginase-1 (Arg-1). This modulation of cytokine levels suppresses neuroinflammation and reduces the infarct area; concurrent *in vitro* experiments showed ATL-III inhibited the JAK2/STAT3 pathway in OGD/R-treated primary microglia, with similar effects on cytokine profiles (Zhou et al., 2019). Research indicates that oral ATL-III administration in MCAO/R rat hippocampus activates the PI3K/Akt pathway while inhibiting the NF-κB pathway and NOD-like receptor family pyrin domain-containing 3 (NLRP3) inflammasome, leading to reduced levels of IL-1β, IL-6, and TNF-α. Similar effects were observed in OGD/R-treated primary microglia (Mao et al., 2026). *In vitro* studies demonstrate that ATL-III reduced OGD/R-induced elevated levels of IL-1β, IL-6, and TNF-α in HT22 cells (Guo et al., 2024).

BBB impairment is a key factor in post-stroke brain injury. According to Mao et al. (2026) (Mao et al., 2026), oral ATL-III enhances the expression of zonula occludens-1 (ZO-1), occludin, and claudin-1 in the hippocampus of MCAO/R rats, aiding in the repair of the BBB.

Sonic hedgehog (Shh) is a secreted protein crucial for neural development and cell growth. Shh interacts with its receptor Patched 1 (Ptch1), triggering the activation of the transcription factor glioma-associated oncogene homolog 1 (Gli1), which subsequently initiates the expression of genes associated with cell cycle progression (Zhou et al., 2022). Upregulating the Shh/Ptch1/Gli1 pathway exerts beneficial repair-promoting effects on ischemic injury (Costa et al., 2023). Studies show oral ATL-III upregulated Shh and Ptch1 expression in the ischemic hemisphere of MCAO/R rats and reduced infarct area (Zhou and Lu, 2024). However, another *in vitro* study reported contrary results, finding ATL-III downregulated Shh and Gli1 expression in HT22 cells, inhibiting OGD/R-induced apoptosis, inflammation, and oxidative stress (Han et al., 2025).

### 5.3 Effects of ATL-III on depressive disorder models

The World Health Organization states that depression is now the third most significant contributor to the global disease burden and is projected to be the top cause by 2030 (Malhi and Mann, 2018). The COVID-19 pandemic led to a rise in global major depressive disorder (MDD) cases, with surveys showing that nearly 40% of Chinese adults experienced depressive symptoms during this period (Zeng et al., 2024).

Depression onset is closely linked to neuroinflammation and neuronal damage. Increased pro-inflammatory cytokines in the brain may cause neuronal atrophy and synaptic dysfunction (Xie et al., 2023). According to Zhou et al. (2021) (Zhou et al., 2021), oral ATL-III alleviated depressive symptoms in both lipopolysaccharide (LPS)-induced and chronic unpredictable mild stress (CUMS) depression models, while also reducing hippocampal TNF-α, IL-1β, and IL-6 levels in CUMS rats. ATL-III mitigated corticosterone-induced apoptosis, inflammation, and mitochondrial dysfunction in PC12 cells *in vitro* by inhibiting the NF-κB and mitogen-activated protein kinase (MAPK) pathways (Gong et al., 2019).

### 5.4 Effects of ATL-III on spinal cord injury

Direct or indirect external force applied to the spinal cord can cause organic damage. Spinal cord injury (SCI) leads to sensory and motor dysfunction, ultimately resulting in varying degrees of paralysis (McGarry, 2025). The SCI process involves primary injury from the initial force and subsequent secondary injury triggered by factors like inflammation (Alizadeh et al., 2019). Although research has advanced considerably, universally effective treatments for SCI are still lacking (Skinnider et al., 2024).

Microglia consistently remove damaged neurons and pathogens in the CNS, ensuring synaptic balance (Frosch et al., 2025). Microglia can activate and differentiate into M1 and M2 subtypes. M1 microglia induce neuroinflammation, whereas M2 microglia have anti-inflammatory effects (Wang H. et al., 2022). Research indicates that oral ATL-III suppresses M1 polarization and encourages M2 polarization of microglia/macrophages by inhibiting NF-κB, c-Jun N-terminal kinase (JNK) MAPK, and P38 MAPK pathways, while activating the Akt pathway. This modulation reduces neuroinflammation and aids in spinal cord repair and motor function recovery in rats’ post-SCI (Xue et al., 2022).

### 5.5 Effects of ATL-III on neuroinflammation and oxidative stress injury models

Several researchers have established *in vitro* inflammation models using LPS to observe ATL-III’s inhibitory effects on neuroinflammation. Cai et al. (2025) (Cai et al., 2025) demonstrated that ATL-III suppresses NF-κB activation in BV-2 cells, leading to reduced phosphorylation of p65 and IκBα, decreased inflammatory cytokines, and improved LPS-induced inflammation and mitochondrial dysfunction. This improvement is achieved by restoring mitochondrial membrane potential and upregulating PPAR gamma coactivator-1α (PGC-1α) and cytochrome C oxidase subunit 4 (COX4) (Cai et al., 2025). Yi et al. (2021) (Yi et al., 2021) demonstrated that ATL-III mitigates inflammation in LPS-stimulated BV2 cells by targeting the toll-like receptor 4 (TLR4)/NF-κB pathway, leading to decreased levels of pro-inflammatory cytokines and cytotoxic agents. ATL-III modulates LPS-activated BV2 cells by inhibiting NF-κB, JNK MAPK, and p38 MAPK pathways while activating the Akt pathway, thereby suppressing M1 polarization and promoting M2 polarization, which leads to reduced expression of associated inflammatory mediators (Xue et al., 2022). ATL-III was shown to downregulate TLR4 expression and inhibit the p38 MAPK, JNK, and NF-κB pathways, leading to reduced production of pro-inflammatory cytokines and enzymes in LPS-stimulated MG6 cells (Novianti et al., 2021). ATL-III decreased IL-1β, IL-6, and TNF-α expression and counteracted LPS-induced autophagy suppression in LPS-stimulated BV2 cells (Zhou et al., 2023).

Furthermore, in an *in vitro* oxidative stress injury model, ATL-III reduced production of ROS, H_2_O_2_, and iNOS while increasing antioxidant capacity (AOC) and SOD production in hydrogen peroxide-damaged Neuro-2A cells (Yuan et al., 2025).

## 6 Discussion

While non-communicable, CNSDs profoundly impact quality of life by not only causing neurological damage but also impairing an individual’s motor, speech, memory, and intellectual functions (Wong et al., 2017). Extracting neuroprotective natural medicinal metabolites from traditional botanical drugs represents a promising drug discovery pathway. In the past decade, research has pointed to ATL-III as a promising candidate for neuroprotection. This is the first review to provide a systematic summary and critical evaluation of ATL-III’s therapeutic potential and mechanisms in various CNSDs.

### 6.1 Summary of the neuroprotective actions and underlying mechanisms of ATL-III in CNSDs

**FIGURE 4:**
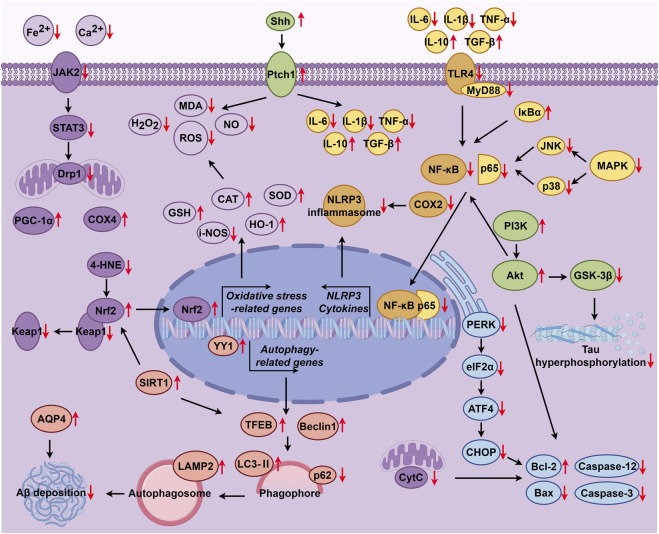
Summary of the molecular mechanisms of atractylenolide III in central nervous system disorders.

The neuroprotective potential of ATL-III against CNSDs, supported by consistent preclinical findings, stems from its multifaceted action across various targets. Its efficacy rests on three interconnected pharmacological pillars: the modulation of oxidative stress, the suppression of inflammation, and the regulation of cellular vitality (Figure 4).

Oxidative stress represents a state of imbalance between oxidation processes and antioxidant defenses. It can significantly disrupt mitochondrial function, causing homeostasis imbalance, mitochondrial fragment release, and increased ROS production. During the development of CNSDs, the brain’s high oxygen consumption makes it prone to oxidative imbalance, generating more ROS; simultaneously, limited antioxidant enzyme function in brain neurons hinders efficient, rapid clearance of free radical-induced damage under oxidative stress. ATL-III exhibits strong antioxidant properties. It modulates the Keap1/Nrf2/HO-1 pathway to enhance antioxidant enzyme activity and eliminate ROS. Conversely, it can suppress mitochondrial fission by regulating the JAK2/STAT3/Drp1 pathway and enhance mitochondrial function through modulation of PGC-1α and COX4.

Controlled inflammation facilitates tissue repair, but when excessive, it exacerbates damage and promotes oxidative stress. Central to neuroinflammation is the activation of glial cells, which release damaging pro-inflammatory factors. ATL-III counteracts this process by modulating key pathways such as PI3K/Akt, NF-κB, JAK2/STAT3, and MAPKs. Mechanistically, it blocks NF-κB cascade activation, inhibits the NLRP3 inflammasome, and diminishes pro-inflammatory cytokine production.

A central component of ATL-III’s neuroprotection is its broad capacity to regulate cellular vitality. This is achieved by promoting cell survival through the inhibition of apoptosis (via PI3K/Akt, MAPKs, and JAK2/STAT3 pathways) and the suppression of ER stress (through the PERK/eIF2α/ATF4 pathway). Furthermore, ATL-III modulates cellular homeostasis by regulating autophagy via the YY1/TFEB and mTOR/Beclin1/LC3-II/P62 axes, which supports the clearance of neurotoxic proteins such as Aβ plaques and neurofibrillary tangles. Beyond intracellular mechanisms, ATL-III also exerts tissue-level protective effects by repairing blood-brain barrier damage through tight junction protein regulation and restoring neural circuit function via the modulation of neurotransmitter levels (e.g., ACh), ultimately contributing to neuronal resilience.

Notably, ATL-III may exhibit bidirectional regulatory effects on certain signaling pathways. For example, it can upregulate the Shh pathway to promote repair after ischemic brain injury, yet also prevent Shh pathway overactivation-induced apoptosis, inflammation, and oxidative stress following OGD/R. It promotes autophagy to clear Aβ, yet also inhibits excessive autophagy to prevent anesthetic-induced neuronal damage.

### 6.2 Critical analysis of current research

Although preclinical findings are encouraging, several limitations and knowledge gaps must be addressed to advance this field and facilitate clinical translation.

First, the indications of ATL-III for CNSDs need to be further expanded. Although its efficacy has been demonstrated in several CNSD models discussed in this review, its potential in other highly prevalent diseases (e.g., Parkinson’s disease, anxiety disorders, or intracerebral hemorrhage) remains unexplored.

Second, the targets and mechanisms of action of ATL-III are still not fully elucidated. On the one hand, most existing studies have focused on classical mechanisms of cellular injury such as oxidative stress and inflammation, as well as classical programmed cell death pathways including apoptosis and autophagy. Less attention has been paid to multi-organ crosstalk (e.g., the gut-brain axis) and novel forms of programmed cell death (e.g., PANoptosis). On the other hand, although ATL-III has been shown to act on a wide range of targets, most studies remain at a phenomenological level, relying on techniques such as Western blotting or quantitative PCR to obtain data at single time points. The temporal dynamics and sequence of regulation of these signaling pathways remain largely unexplored. Moreover, the differential effects of ATL-III on neurons, microglia, and astrocytes have not been thoroughly investigated. Notably, ATL-III exhibits bidirectional regulatory effects (e.g., promoting autophagy to clear toxic proteins vs. inhibiting excessive autophagy to prevent cell death), and the underlying mechanisms are not yet fully understood.

Third, the safety profile of ATL-III remains inadequately assessed. Acute toxicity studies indicate the median lethal dose of *A. macrocephala* Koidz. Extract in rats and mice is ≥ 10 g/kg body weight (Ma et al., 2025); the no-observed-adverse-effect level for sub-chronic toxicity in female and male rats was 1,306 mg/kg and 1,680 mg/kg body weight, respectively (Ma et al., 2025). Micronucleus tests and sperm abnormality tests have indicated no obvious genotoxicity of *A. macrocephala* Koidz. extract at a dose of 10 g/kg body weight (Zhang J. M. et al., 2025). These studies suggest that *A. macrocephala* Koidz. Is relatively non-toxic at the daily dose recommended by the *Chinese Pharmacopoeia*. However, systematic reports on the toxicity, dose-toxicity relationship, and detailed toxicological mechanisms of pure ATL-III are still lacking.

Fourth, studies aimed at enhancing the bioavailability and BBB penetration of ATL-III are lacking. After oral administration of an aqueous extract of *A. macrocephala* Koidz. To rats, ATL-III showed the highest absorption rate among ATL-I, -II, and -III in all intestinal segments (Gao et al., 2018). Following oral administration of 26.8 mg/kg ATL-III to rats, the time to peak (T_max_) was 0.75 h, the half-life (t_1_/_2_) was approximately 2.92 h, and the maximum plasma concentration (C_max_) was 1,211 ng/L (Liu et al., 2024). After oral administration of 100 mg/kg ATL-III, T_max_ was 0.85 h, t_1_/_2_ was 2.84 h, the apparent volume of distribution was 5.48 L/kg, and the total plasma clearance was 4.63 L/(hkg) (Li et al., 2006). These pharmacokinetic studies indicate that ATL-III is rapidly absorbed and quickly reaches distribution equilibrium *in vivo*, but is also eliminated rapidly, with a moderate bioavailability. After oral administration of 100 mg/kg ATL-III to rats, the highest distribution was observed in the lungs, followed by the cerebellum, heart, and brain, and the compound was mainly eliminated via the spleen, followed by the liver and kidneys (Li et al., 2006). ATL-III was detectable in rat brain microdialysate after oral administration of *Chaigui* Granules, confirming its BBB permeability and potential for central effects (Gao et al., 2020). Although ATL-III can cross the BBB, its brain-to-plasma concentration ratio and brain exposure remain unclear. Both pharmacodynamic and pharmacokinetic studies support the clinical development potential of ATL-III. However, maximizing the bioavailability, BBB penetration, and especially the brain retention time of ATL-III to meet clinical application needs remains an urgent and unresolved issue.

Finally, although there are diverse *in vivo* mechanistic studies on the neuroprotective activity of ATL-III, the lack of relevant clinical trials is a major limitation that urgently requires further investigation. Clinical studies have shown that Chinese herbal formulas containing *A. macrocephala* Koidz. Exert significant therapeutic effects on CNSDs, providing valuable indirect evidence for the potential utility and safety of ATL-III in patients. Nevertheless, clinical studies using pure ATL-III for the treatment of CNSDs are urgently needed to provide more direct evidence for translational medicine.

### 6.3 Future perspectives

First, the therapeutic scope of ATL-III needs to be further explored. Actively investigating the potential of ATL-III in the prevention and treatment of CNSDs such as Parkinson’s disease, anxiety disorder and intracerebral hemorrhage, and further expanding its indications, is an important direction for future research.

Second, unknown targets of ATL-III need to be further explored and known targets need to be validated. Future studies should integrate advanced methods such as cell-type-specific knockout models, multi-omics, and bioinformatics from multiple perspectives including multi-organ crosstalk, dynamic target regulation, and dose-effect thresholds, to fully elucidate and validate the targets of ATL-III and their interactions, and to draw a more accurate map of the regulatory mechanisms. This will enable the systematic construction of a temporal-spatial regulatory network of “target-pathway-disease” and “target-cell type-organ” for ATL-III, further clarifying its mechanism of action against neurological diseases.

Third, the safety profile of ATL-III must be rigorously and comprehensively evaluated. Systematic reports on the toxicity, dose-toxicity relationship, and detailed toxicological mechanisms of pure ATL-III compound require further in-depth investigation.

Fourth, studies on the bioavailability and BBB penetration of ATL-III must be strengthened. Integrating modern pharmaceutical technologies, advanced excipients, and biomaterials to enhance the stability of ATL-III, and synergistically optimizing its bioavailability, BBB targeting, and brain retention, is a key and challenging direction for future research. The ultimate goal is to develop a quality-controllable, highly efficient, and stable ATL-III formulation suitable for clinical application.

Finally, clinical trials must be conducted to provide direct evidence for the use of ATL-III in treating CNSDs. In the future, conducting rigorous, high-quality, large-scale, multicenter randomized controlled trials is essential to validate the efficacy and safety of ATL-III.

### 6.4 Limitations of this review

It is important to note that this review prioritized studies utilizing CNS-relevant cellular models. Consequently, findings from non-neural *in vitro* systems were excluded. Although this ensures biological relevance, it introduces a constraint; the excluded data, while valuable for elucidating fundamental molecular interactions, cannot inform on systems-level functional recovery. This limitation should be considered when evaluating the comprehensiveness of the mechanistic profile presented for ATL-III.

## 7 Conclusion

CNSDs, with their complex etiologies, constitute a major health challenge. ATL-III has demonstrated considerable preclinical promise for disorders like AD, MDD, CIS, and SCI via its multi-target engagement, establishing a strong foundation for its neuroprotective candidacy. However, incompletely elucidated targets and mechanisms, inadequately assessed safety profile, unsatisfactory bioavailability, BBB penetration, and brain retention time, as well as the lack of clinical trials, limit the clinical prospects of ATL-III. In future research, key breakthroughs are needed in expanding the CNSD indications of ATL-III, elucidating its targets and mechanisms, clarifying its safety profile, improving its bioavailability, BBB penetration, and brain retention time, and conducting large-scale clinical studies.

## Glossary

4-HNE: 4-hydroxynonenal
Ach: Acetylcholine
AChE: Acetylcholinesterase
ATF4: Activating transcription factor 4
AD: Alzheimer’s disease
APP: Amyloid precursor protein
Aβ: Amyloid-beta
AQP4: Aquaporin 4
Arg-1: Arginase-1
ATL-III: Atractylenolide III
Bcl-2: B-cell lymphoma-2
Bax: Bcl-2 associated X protein
BBB: Blood-brain barrier
CHOP: C/EBP-homologous protein
CAT: Catalase
CNSDs: Central nervous system disorders
CNS: Central nervous system
CIRI: Cerebral ischemia/reperfusion injury
CUMS: Chronic unpredictable mild stress
JNK: c-Jun N-terminal kinase
Caspase-12: Cysteinyl aspartate-specific proteinase 12
Caspase-3: Cysteinyl aspartate-specific proteinase 3
COX4: Cytochrome C oxidase subunit 4
Drp1: Dynamin-related protein 1
ER: Endoplasmic reticulum
eIF2α: Eukaryotic initiation factor 2α
Gli1: Glioma-associated oncogene homolog 1
GSH: Glutathione
GSK-3β: Glycogen synthase kinase-3β
t1/2: Half-life
HO-1: Heme oxygenase-1
iNOS: Inducible nitric oxide synthase
IL-10: Interleukin-10
IL-1β: Interleukin-1β
IL-6: Interleukin-6
JAK: Janus kinase
Keap1: Kelch-like ECH-associated protein 1
LDH: Lactate dehydrogenase
LPS: Lipopolysaccharide
LAMP2: Lysosome-associated membrane protein 2
MDD: Major depressive disorder
MDA: Malondialdehyde
mTOR: Mammalian target of rapamycin
Cmax: Maximum plasma concentration
LC3-II: Microtubule-associated protein 1 light chain 3-II
MCAO/R: Middle cerebral artery occlusion/reperfusion
MAPK: Mitogen-activated protein kinase
NLRP3: NOD-like receptor family pyrin domain-containing 3
Nrf2: Nuclear factor erythroid 2-related factor
NF-κB: Nuclear factor kappa-B
OGD/R: Oxygen-glucose deprivation/reoxygenation
Ptch1: Patched 1
PI3K: Phosphatidylinositol 3-kinase
PERK: PKR-like endoplasmic reticulum kinase
PGC-1α: PPAR gamma coactivator-1α
PS1: Presenilin 1
Akt: Protein kinase B
ROS: Reactive oxygen species
STAT: Signal transducer and activator of transcription
SIRT1: Sirtuin 1
Shh: Sonic hedgehog
SCI: Spinal cord injury
STZ: Streptozotocin
SOD: Superoxide dismutase
Tmax: Time to peak
TLR4: Toll-like receptor 4
TCM: Traditional Chinese Medicine
TFEB: Transcription factor EB
TNF-α: Tumor necrosis factor α
YY1: Yin Yang 1
ZO-1: Zonula occludens-1
